# Multiple Sclerosis, Cannabis Use, and Clinical Disability: A Preliminary [^18^F]-Fluorodeoxyglucose Positron Emission Tomography Study

**DOI:** 10.1089/can.2018.0019

**Published:** 2018-10-13

**Authors:** John H. Kindred, Justin M. Honce, Jennifer J. Kwak, Thorsten Rudroff

**Affiliations:** ^1^ Department of Health and Exercise Science, Colorado State University, Fort Collins, Colorado.; ^2^ Division of Physical Therapy, Medical University of South Carolina, Charleston, South Carolina.; ^3^ Ralph H. Johnson Veteran's Administration Medical Center, Charleston, South Carolina.; ^4^ Department of Radiology, University of Colorado School of Medicine, Aurora, Colorado.; ^5^ Department of Health and Human Physiology, University of Iowa, Iowa City, Iowa.

**Keywords:** marijuana, motor function, cognitive function, glucose uptake

## Abstract

**Introduction:** Long-term consequences of medicinal cannabis use in people with multiple sclerosis (PwMS) are unknown. This study investigated whether PwMS using cannabis had lower resting brain glucose uptake (GU) and worse clinical test results compared with nonusers.

**Methods:** Sixteen PwMS, eight users, underwent clinical testing followed by [^18^F]-Fluorodeoxyglucose positron emission tomography/computed tomography imaging.

**Results:** Users had lower cognitive function test scores, but performed similarly on the other clinical evaluations. Accounting for disease duration, resting brain GU was similar between the groups.

**Conclusions:** Lower cognitive function was not associated with resting brain GU. Cognitive dysfunction may be a contraindication or consequence of cannabis use in PwMS.

## Introduction

One of the major hurdles to medical cannabis acceptance or rejection is that benefits and consequences of long-term cannabis use in clinical populations has not been established. Recent estimates show 20–66% of people with multiple sclerosis (PwMS) are currently using cannabis,^1,2^ even though there is little empirical evidence showing its efficacy. Several short-term interventional studies have generally shown some beneficial effects on spasticity and pain,^3,4^ and have led to loose recommendations for cannabis use.^5^ While some benefits have been observed, several studies reported that PwMS using cannabis performed worse on cognitive tasks.^6–8^

Positron emission tomography (PET), with the glucose analog [^18^F]-Fluorodeoxyglucose (FDG), has been used to investigate brain function in healthy and clinical populations. FDG-PET studies have shown that regular heavy alcohol use,^9^ cannabis,^10^ and other illegal drug use^11,12^ could lead to lower resting nervous system glucose uptake (GU). It has been suggested that lower brain GU may be responsible for some of the cognitive and physical impairments seen in these drug abuse populations.^10,11^ In PwMS, lower brain GU has been associated with clinical symptoms, such as: fatigue,^13^ cognitive ability,^14^ and walking speed.^15^ Brain GU may be an effective biomarker for the tracking of disease progression and disability in PwMS. How cannabis use affects brain GU in PwMS is unknown.

The purpose of this preliminary investigation was to measure and compare resting brain GU in PwMS regularly using and not using cannabis. Based on findings from Volkow et al.,^10^ our *a priori* hypothesis was that GU would be lower in cannabis users compared with nonusers. We further hypothesized that cannabis users would perform worse on clinical tests, and that performance would be linked to reduced GU in the cannabis users, providing insight into possible mechanisms of dysfunction.

## Materials and Methods

### Ethical statement

All procedures were approved by the local institutional review boards and conformed to the Declaration of Helsinki. All participants signed informed consent before participating in the study.

### Participants and clinical tests

Sixteen PwMS, eight cannabis users and age/sex-matched nonusers, underwent clinical testing and FDG-PET/computed tomography (CT) imaging. Participants with relapses within the past 3 months, concurrent neurologic disorders, and resting plasma glucose >200 mg/dL were excluded. Only participants using cannabis for at least 6 months were included. Participants refrained from cannabis and fasted, 8 and 4 h respectively, before arrival at the Colorado Translational Research Imaging Center (Denver, CO). Disability levels were evaluated using the Patient Determined Disease Steps (PDDS).^16^ A urinalysis was conducted on all participants to detect the presence of Δ9- THC (iScreen IS1THC dipstick; Alere Toxicology, MA). Clinical testing consisted of the following: Activities of Balance Confidence,^17^ Beck's Depression Inventory,^18^ Fatigue Severity Scale,^19^ Pain Effects Scale, Numerical Rating Scale measure of spasticity,^20^ Multiple Sclerosis Functional Composite (MSFC),^21^ handgrip strength testing, timed up-and-go,^22^ and balance testing.

Handgrip strength was measured using a hand dynamometer (Lafayette Instruments, IN).^23^ Maximal force output, corrected for total body weight, of both hands was averaged for the analysis. For balance testing, participants standing quietly, unshod, for 30 sec, eyes open, with the medial malleoli and metatarsal/phalangeal joints of each foot touching. Center of pressure movement was tracked using a BTrackS balance board (Balance Tracking Systems, Inc., CA). The MSFC consisted of the 25-foot walk test, nine-hole peg test, and Paced Auditory Serial Addition Test (PASAT). For the 25-foot walk, participants were timed while walking 25 feet. This was done twice with the quickest time used in the analysis. The nine-hole peg test was performed twice with each hand. The time it took for the participants to place nine pegs into a 3×3 grid, and then remove them was recorded. The lowest time was used for analysis. During the PASAT participants added two prerecorded numbers presented at a rate of one digit every 3 sec for 3 min. The number correct was compared between the groups. Participants tracked their cannabis use in a log for 7 days and provided available cannabis product labels listing the THC and cannabidiol content to the investigators.

### Image acquisition

Blood glucose concentrations were measured via finger stick. Nine mCi of FDG was injected into an intravenous catheter. Participants rested quietly during the 35-minute radiopharmaceutical uptake period. Imaging was performed on a Hybrid Gemini TF64 (Philips Healthcare, OH). An initial CT scan of the head was performed for localization and attenuation-correction (120 kV, 100 mAs, 2 mm slice thickness) followed by a one-bed PET scan of the head. After 45 min of tracer uptake, 10 min of PET list-mode data was initiated. Attenuation-corrected images were reconstructed using the three-dimensional row-action maximum likelihood algorithm (RAMLA) method.

### Image analysis

Image preprocessing was performed using Analyze 11.0 (Mayo Clinic, Rochester, MN). CT attenuation-corrected PET images were converted into standardized uptake value (SUV) images^24^ and exported as Analyze 7.5 files. SUV images were spatially normalized to Montreal Neurological Institute space and smoothed to an FDG template within SPM12 (The Wellcome Trust Centre for Neuroimaging, London, United Kingdom).^25^ SUV images were corrected for tracer radioactive decay and participant body weight.

An unpaired t-test, with covariate of disease duration, was performed within SPM12 (relative threshold masking=0.8). Alpha equaled 0.05 and an extent threshold (k_e_)=50 (voxels) was used. Mean SUVs for brain regions were extracted using the Marsbar SPM toolbox.^26^ Regions included: anterior and posterior cerebellar lobes; frontal, temporal, occipital, and parietal lobes; medulla, midbrain, and pons.^10^

### Statistical analysis

All data are reported as mean (standard deviation) unless otherwise noted. Pearson's correlations were calculated between demographic variables (age, height, weight, body mass index [BMI], disease duration) and whole brain SUV to identify possible covariates for the SPM analysis. Significant factors were individually tested as covariates during SPM analysis.

Demographic, clinical, and extracted reginal SUV variables were compared between the two groups using unpaired t-tests. If a clinical variable differed between the two groups a Pearson's correlation was performed with regional SUVs. Cohen's D measures of effect size were calculated for regional SUVs. Analysis was performed using IBM SPSS Statistics for Windows version 24 (IBM Corp., NY) with alpha set to 0.05.

## Results

The age of the participants (*n*=16) was 50.2(SD 13.6) years with a disease duration of 11.7(SD 8.3) years. The median disability level was 2(range 0–6). Disease duration was not different between the groups (*p*=0.12), however, it correlated with whole brain SUV (*R*=−0.63, *p*=0.01) and was subsequently used as a covariate during SPM analysis. No other demographic variables correlated with whole brain SUV (Age, *R*=0.00, *p*=1.00; height, *R*=−0.36, *p*=0.18; weight, *R*=0.00, *p*=0.99; BMI, *R*=0.41, *p*=0.12; PDDS, *R*=−0.39, *p*=0.14).

Seven of eight cannabis users had been using for >12 months, and one for 6 months. Users reported ingesting cannabis 2.3 (SD 1.5) times/day and 6.9 (SD 0.4) days/week. Cannabis usage was mostly in the form of edibles and smoking. Seven users returned at least one product label to the investigators. Cannabis users used 2.4 (SD 1.8) different products. Product labels that were returned to the research team from the THC dominant products often did not list the CBD content. Three users primarily used CBD dominant products (CBD:THC >5:1) (Supplementary Table S1). Each user, regardless of using THC or CBD dominant products, tested positive and all nonusers tested negative for THC via urinalysis.

Comparisons for demographic, questionnaires, and functional variables were similar (*p*>0.12), except cannabis users had lower PASAT scores (*p*=0.02) (Table 1). PASAT scores did not correlate with regional SUVs (*R* < −0.35, *p*>0.18) (Table 2).

**Table 1.** **Demographics and Performance Test Values**

	Nonusers	Users	*p*
Sex (M/F)	2/6	2/6	
Age (years)	50.8 (13.2)	49.6 (15.0)	0.875
Ht. (m)	1.7 (0.1)	1.7 (0.1)	0.186
Wt. (kg)	79.0 (25.5)	75.7 (20.0)	0.521
BMI	25.8 (5.4)	26.7 (5.2)	0.739
Disease duration (years)	14.9 (8.3)	8.4 (7.4)	0.121
PDDS	2.1 (1.8)	2.4 (1.8)	0.788
Handgrip strength (kg/kg bw)	0.46 (0.13)	0.37 (0.08)	0.122
25Ft Walk Test (sec)	6.2 (4.8)	5.8 (2.1)	0.855
PT (Dom) (sec)	23.1 (6.6)	24.2 (9.4)	0.798
PT (Non) (sec)	22.7 (5.1)	24.7 (7.8)	0.570
PASAT (number correct)	43.5 (8.8)	31.8 (8.6)	0.018^*^
COP movement (% Ht.)^a^	0.28 (0.06)	0.36 (0.09)	0.095
TUG (sec)	12.1 (14.3)	9.7 (4.1)	0.644
ABC	78.5 (18.6)	69.5 (26.1)	0.437
BDI	12.6 (7.1)	14.1 (6.1)	0.657
FSS	4.9 (2.3)	5.3 (1.3)	0.690
PES	13.9 (6.8)	16.1 (6.4)	0.506
NRS	3.6 (4.0)	1.9 (1.8)	0.282

Comparisons between the groups were made with unpaired student's *t*-tests.

^*^ Significant.

^a^ *n*=7 nonusers; *n*=8 users.

ABC, Activities of Balance Confidence; BDI, Beck's Depression Inventory; BMI, body mass index; BW, body weight; COP, center of pressure; Dom, dominant; FSS, Fatigue Severity Scale; Non, nondominant; NRS, Numerical Rating Scale of Spasticity; PASAT, Paced Auditory Serial Addition Test; PDDS, Patient Determined Disease Steps; PES, Pain Effects Scale; PT, 9-hole peg test; TUG, timed up and go.

**Table 2.** **Correlation Analysis Between Paced Auditory Serial Addition Scores and Brain Standardized Uptake Values**

*n*=16	Pearson's correlation	*p*
Whole brain	−0.353	0.180
Cerebellum (Ant)	0.013	0.962
Cerebellum (Post)	−0.002	0.994
Frontal lobe	0.048	0.860
Occipital lobe	0.009	0.973
Parietal lobe	−0.002	0.994
Temporal lobe	−0.009	0.972
Medulla	0.129	0.634
Midbrain	0.102	0.708
Pons	0.102	0.707

Only the PASAT was different between the cannabis users and nonusers but the scores from this assessment were not correlated with brain glucose uptake.

SPM12 analysis revealed areas throughout the brain, primarily within the frontal and parietal lobes, in which cannabis users had greater GU, although these results did not persist when disease duration was taken into account. Mean SUVs for the groups/regions were not different. Effect sizes and SUVs are listed in Table 3.

**Table 3.** **Average Standardized Uptake Values for Whole and Regional Brain Areas and Calculated Effect Sizes**

	Nonusers	Users	*p*	Cohen's D
Whole brain	5.2 (0.8)	5.7 (0.9)	0.33	0.50
Cerebellum (Ant)	6.6 (1.0)	7.2 (1.5)	0.38	0.45
Cerebellum (Post)	6.7 (0.9)	7.2 (1.6)	0.42	0.41
Frontal lobe	7.4 (1.4)	8.0 (1.5)	0.36	0.47
Occipital lobe	8.4 (1.6)	9.3 (2.0)	0.30	0.53
Parietal lobe	8.1 (1.5)	9.0 (1.9)	0.31	0.52
Temporal lobe	6.9 (1.3)	7.7 (1.4)	0.26	0.58
Medulla	5.8 (1.0)	6.1 (1.4)	0.57	0.29
Midbrain	6.4 (1.1)	6.8 (1.2)	0.51	0.34
Pons	5.0 (0.8)	5.4 (1.1)	0.31	0.37

Unpaired student's *t*-tests were performed to determine whether values differed between the users (*n*=8) and nonusers (*n*=8).

Ant, anterior; Post, posterior.

## Discussion

This is the first investigation reporting resting brain GU of PwMS regularly using cannabis. Our main findings: Regular cannabis use does not result in lower resting brain GU in PwMS, balance and mobility were mostly similar between the groups, and PASAT scores were lower in cannabis users. Lower PASAT scores were not associated with lower GU.

### Resting brain GU

In healthy cannabis users, several studies have shown lower global and regional GU suggesting a neuroadaptive process to regular cannabis use, possibly related to downregulation of the CB1 receptors due to regular THC exposure.^10,27^ This possible scenario is supported by the apparent normalization of brain activity with prolonged abstinence.^27^ Reduced brain GU in the cannabis users could account for the impairments in motor performance reported in these subjects.^28,29^ However, reduced brain GU was typically associated with minimal or no change in behavioral performance during task performance in imaging studies.^30,31^

Several studies have shown lower brain GU in PwMS.^13–15,32^ Reduced GU in gray and white matter may relate to direct or indirect effects of MS lesions.^14,32^ In this study we did not detect lower GU in cannabis users. This would appear to indicate a different neuroadaptive process in the MS brain compared with non-MS brains. An integral factor to nervous system GU is circulating levels of lactate. PwMS have been shown to have increased levels of lactate, which is thought to stem from increased inflammation and neurodegeneration within the central nervous system.^33^ Smith et al.^34^ showed that infusing lactate peripherally lowered nervous system GU in healthy individuals. Cannabis use in PwMS may lead to lowered inflammation/neurodegeneration,^35,36^ and thereby have an offsetting effect between speculated CB1 downregulation and reduced nervous system inflammation, leading to a zero-net change in GU.

### Cognitive function and cannabis use in multiple sclerosis

Cannabis use has previously been negatively associated with cognitive function in PwMS.^6–8^ Romero et al.^6^ utilized MRI to investigate morphological effects on cognitive function in cannabis users and nonusers with MS. They found that regional volumes correlated with cognitive performance, although the regions and the strength of the correlations differed between the users and nonusers. For example, white matter volume correlated only with tests involving processing speed in noncannabis using PwMS, while this metric correlated to all cognitive measures in cannabis users. This appears to indicate a maladaptive process in the cannabis users, requiring additional brain circuit usage to perform cognitive tasks. In this study, we found no association between resting activity of any region and PASAT scores, and correlations performed for each individual group (data not reported) also did not indicate an association between the variables. Our findings suggest that cannabis use may have deleterious effects on cognitive performance, as evidenced by the lower PASAT scores. However, the mechanism behind this possible effect is still unknown.

### Limitations

Two prominent limitations of this study are the cross-sectional design and the small sample size. Our findings suggest that long-term cannabis use does not negatively affect brain GU. However, the cross-sectional nature of this study does not let us determine whether brain GU would be different in regular users if they were asked to be abstinent for a longer period. The small sample size also reduces the generalizability of our results, and larger interventional and/or longitudinal studies are certainly required.

Most of the product labels collected during the study reported much greater amounts of THC compared with CBD. It is generally thought that CBD would have the most therapeutic value due to its nonpsychoactive properties.^35,37^ In this study, all users tested positive for THC via urinalysis, but circulating blood levels of THC, its derivatives, and CBD may provide additional insight into nervous system function and cannabis use.

## Conclusions

This study did not detect any effects of regular cannabis use on resting brain GU, which has been shown to be a marker for clinical disability.^13–15^ The lower cognitive performance in PwMS currently using cannabis is consistent with the current literature.^6–8,38–40^ However, the mechanisms of this dysfunction have yet to be elucidated. Based on our preliminary findings, it is unlikely that resting brain metabolic activity/GU account for the differences in cognitive ability, assessed by the PASAT measure. As stated in a previous review,^40^ there is a crucial need to determine the effects of commercially available cannabis products to increase the knowledge of risks and benefits of cannabis use in PwMS.

## Supplementary Material

Supplemental data

## Abbreviations Used

BMI: body mass index
CBD: cannabidiol
CT: computed tomography
FDG: fluorodeoxyglucose
GU: glucose uptake
MSFC: Multiple Sclerosis Functional Composite
PASAT: Paced Auditory Serial Addition Test
PDDS: Patient Determined Disease Steps
PET: positron emission tomography
PwMS: people with multiple sclerosis
SUV: standardized uptake value
THC: tetrahydrocannabinol
