# Supplementary material for: Multiple Sclerosis, Cannabis Use, and Clinical Disability: A Preliminary [18F]-Fluorodeoxyglucose Positron Emission Tomography Study
Source: Cannabis Cannabinoid Res. 2018 Oct 13;3(1):213–8. doi: 10.1089/can.2018.0019 (PMC6186162; doi:10.1089/can.2018.0019)
Supplement: Supplemental data [file Supp_Table1.pdf]

Supplementary Data

Supplementary Table S1. Individual Cannabis Use Characteristics of the Sample

ID	Length of use (months)	Days per week	Times per day	THC content/serving	CBD content/serving
01	> 12	7	1	10 mg	—
02	> 12	7	1.7	50 mg	< 0.1 mg
03	> 12	7	5.3	—	—
04	6	7	1	2 mg	10 mg
05	> 12	6	1.7	10 mg	< 0.1 mg
06	> 12	7	3.9	18.24%	0.00%
07	> 12	7	1.4	1 mg	20 mg
08	> 12	7	2	< 0.03	30 mg

THC and CBD contents are reported from the product labels that were returned to the investigators. Some labels did not list any CBD content. Products with "%" refer to smoked flowers.

"—" means value was not reported via label or participant.
